# Navigating in clutter: how bumblebees optimize flight behaviour through experience

**DOI:** 10.1242/jeb.250514

**Published:** 2025-07-25

**Authors:** Manon Jeschke, Maximilian Stahlsmeier, Martin Egelhaaf, Olivier J. N. Bertrand

**Affiliations:** Neurobiology, Bielefeld University, Universitätstr. 25, 33615 Bielefeld, NRW, Germany

**Keywords:** Insect navigation, Cluttered environment, Collision avoidance, Learning, *Bombus terrestris*

## Abstract

Bumblebees are excellent navigators that travel long distances while following paths to known locations. They forage not only in open terrain but also in cluttered environments where obstacles force them to deviate from direct paths. This study investigated the underexplored aspect of how bees become experienced foragers and optimize flight behaviour in cluttered terrains. We recorded flight trajectories of novice bees with no prior experience in navigating cluttered laboratory environments and monitored their behavioural performance as they gained experience on subsequent foraging trips through numerous obstacles. By controlling for experience levels, we analysed how flight characteristics evolve with increasing expertise. Successful navigation in cluttered terrain requires avoiding collisions with obstacles. This is only possible if these can be detected through visual features such as the retinal displacement of contrast edges. Obstacles which are harder to detect and to avoid by the bees may affect their flight performance. By introducing transparent objects into our dense environment, we challenged collision avoidance and learning mechanisms, analysing the impact on flight optimization under different environmental conditions. Our findings reveal that experienced bees fly similar paths through clutter and quickly adapt their flights regardless of their training environment. However, the specific paths followed are influenced by environmental conditions. Transparent objects primarily affect naive bees' flight patterns while having minimal impact on flight optimization, suggesting that the efficient flights of experienced bees result not solely from reflexive collision avoidance but from learning and previous experience in cluttered environments.

## Teaser

**Highlighted Article:** Bumblebees optimize flight paths through cluttered environments via learning rather than reflexive collision avoidance, with experienced foragers quickly adapting routes while transparent obstacles primarily challenge naive bees.

## INTRODUCTION

Navigating to a desired location is a crucial task in everyday life for animals across all taxa. Navigation takes place over a wide range of distances and can be accomplished by using different guiding systems (Buehlmann et al., 2020) and following different motivations, whether the animals are looking for a mating partner (Stepien et al., 2020), performing long-distance migration (Chowdhury et al., 2021) or finding the way home (Gagliardo, 2013).

Bumblebees are skilful navigators and essential pollinators that play a crucial role in maintaining biodiversity and keeping the ecosystem functioning (Goulson et al., 2008; Potts et al., 2010). As central-place foragers, they regularly leave their nest to search for food sources and bring nectar and pollen back to their hive, requiring them to not only memorize nearby feeding sites but also retrace their path and return to a previously visited location. During their foraging flights, these insects often navigate through complex, cluttered environments such as densely vegetated forests, as well as artificial environments such as tomato fields in a greenhouse, where the animal must deviate from a straight path to avoid collisions without drifting away from its overall goal direction (Crall et al., 2015). Efficient collision avoidance and navigation in these challenging environments are critical for the bumblebees' foraging success, as collisions can cause injury or damage to their wings and therefore worsen their flight performance (Foster and Cartar, 2011; Mountcastle et al., 2016).

Previous studies have shown that insects, including bumblebees, rely on visual cues and optic flow for navigation and collision avoidance in complex environments (Baird et al., 2010; Chakravarthi et al., 2017; Lecoeur et al., 2019; Linander et al., 2015). In contrast to walking animals, which can use tactile information to detect obstacles, flying insects such as bees rely on visual information from their eyes to detect and avoid obstacles (Ravi et al., 2019). The visual system of bumblebees is well adapted to their foraging needs, with a relatively high spatial resolution in the frontal region of their eyes and the ability to perceive a wide range of colours (Dyer et al., 2008; Spaethe and Chittka, 2003).

Several studies have investigated the flight characteristics of experienced bumblebees in environments with varying numbers of objects (i.e. different object densities). These studies have shown that bumblebees adapt their ground speed and axial velocity when they encounter obstacles (Burnett et al., 2020; Crall et al., 2015) and that their lateral position varies as they fly through an obstacle field (Burnett et al., 2022). However, navigating in complex environments includes more than just reflexive reactions to avoid collisions with obstacles, which are mostly independent of experience. Learning and establishing a route memory are also key factors for bumblebees to optimize their flight (Burnett et al., 2022; Gonsek et al., 2021). Nevertheless, these studies focused on the flight behaviour of bees experienced with their specific cluttered environment. Therefore, it is not easy to deduce whether the flight around an obstacle is the consequence of reflexive collision avoidance or of previous experience with the cluttered terrains. Thus, the consequences of increasing experience and its interaction with collision avoidance in bumblebees, as well as how these factors contribute to the optimization of their flight behaviour, are still poorly understood.

To gain deeper insight into how collision avoidance and learning mechanisms interact to help bumblebees (*Bombus terrestris*) optimize their flight in dense environments, we recorded their flight behaviour in appropriate laboratory settings while carefully controlling for prior experience. We trained bees in two different environments containing either exclusively dark objects or partly transparent objects to challenge their collision avoidance and learning mechanisms, and to better understand how bumblebees adapt to different visual environments with increasing experience.

To the best of our knowledge, our study is the first to investigate the effect of experience on the optimization of flight characteristics in bumblebees navigating cluttered environments. As bees are able to optimize their journey when collecting food from several spatially distributed sources on a single foraging trip (e.g. traplining in bumblebees; Lihoreau et al., 2010), we hypothesized that bumblebees will also optimize their flight behaviour with increasing experience in cluttered environments. However, the presence of transparent objects in the environment may increase the time the bees need to learn a route and optimize their flight, as these objects are less salient and therefore harder to detect. Our findings contribute to a better understanding of how bumblebees become efficient foragers and navigate in cluttered environments, while simultaneously highlighting the role of both collision avoidance and experience in studies of insect navigation.

## MATERIALS AND METHODS

### Experimental setup

**Fig. 1.**
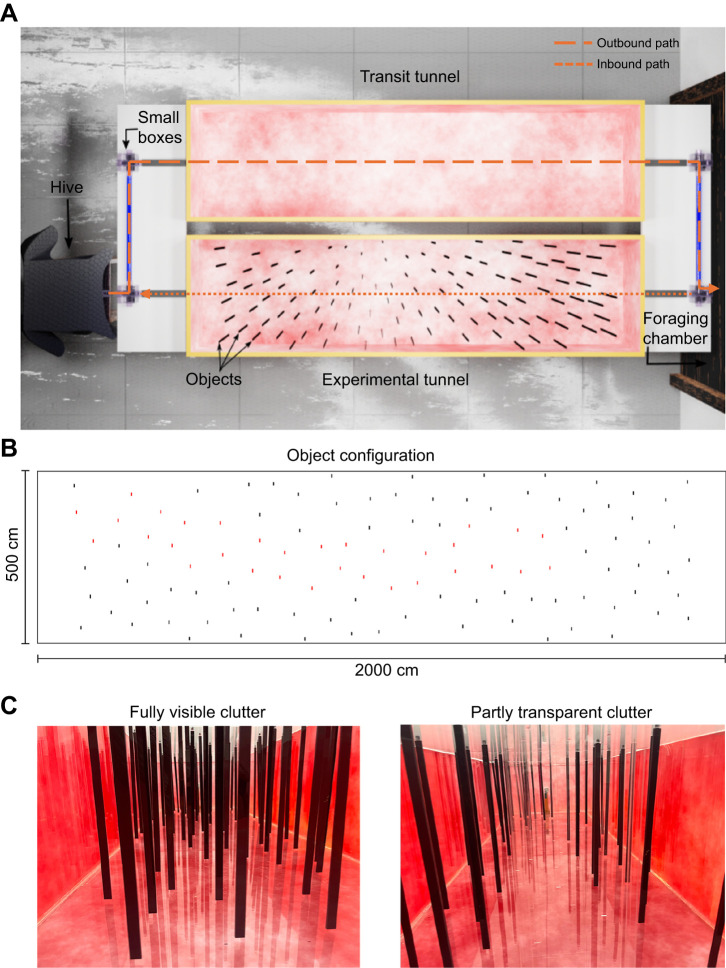
**The experimental setup**. (A) Rendered top view of the setup that was recreated using the graphics software *Blender* (http://www.blender.org). The hive was connected to the setup via small transparent boxes and tubes. Bees entered the foraging chamber (not shown) by flying through the transit tunnel (top). For the experiments, returning foragers were redirected into the experimental tunnel (bottom) where 110 vertical objects formed a complex cluttered environment the bees had to cross to return to the hive. The path of a marked forager through the setup is indicated by arrows. (B) Object configuration. In the second environment, 32 objects (marked in red) were replaced with objects made from transparent acrylic. (C) Photographs taken from within the cluttered environment equipped with either all red objects (left) or transparent objects (right).

The experimental setup consisted of two flight tunnels, connecting the bumblebee hive and a foraging chamber (see Fig. 1A). Bumblebees exiting the hive accessed the flight tunnel for population transit through transparent tubes measuring 2.5 cm in diameter, alongside small acrylic boxes with dimensions of 8×8×8 cm^3^. The flight tunnel's dimensions were 200×50×30 cm^3^. Notably, the walls and floor of the flight tunnels were covered with a red and white 1/*f* noise pattern, as previously described by Ravi et al. (2019), to provide the bees with a pattern characterized by a natural spatial frequency spectrum. Transparent acrylic was utilized for the tunnel ceiling to facilitate the observation of bee behaviour.

Upon their return from the foraging chamber, foragers were rerouted into the experimental tunnel for individual training. This tunnel was equipped with 110 randomly positioned rectangular cuboids, each measuring 29.5×1×0.3 cm^3^, thereby creating a cluttered environment. These objects had a minimal distance of 7 cm to each other to ensure that bees could pass the gaps without rotating along the yaw axis (Ravi et al., 2019). Notably, these objects were made from either transparent or red acrylic, with the latter designed to block light below 650 nm, as verified by spectrometer measurements (see Fig. S1), and therefore appeared dark to the bee (Dyer et al., 2008). The room and the flight tunnels were illuminated by natural daylight entering through the windows in the side walls of the laboratory. To capture the bee's behaviour, four synchronized high-speed cameras (Basler acA2040-90um NIR) equipped with red filters (Heliopan RG715) and 16 mm lenses (Kowa LM16HC) were positioned above the tunnel at different angles. Two cameras provided top-down views from a height of 2 m, each covering one half of the tunnel with a 25 cm overlap. Two other cameras were positioned at the right and left ends of the experimental tunnel at a height of 1.8 m and angled at 45 deg, providing side views down the length of the tunnel with overlap in the centre. After positioning the cameras, we calibrated them to determine the intrinsic parameters (focal length, centre and distortions) and the extrinsic parameters (orientations and positions of the cameras) using a standard checkerboard calibration method and OpenCV software (https://docs.opencv.org/4.x/dc/dbb/tutorial_py_calibration.html).

To improve the visibility of the bees in the video recordings, the tunnel was additionally illuminated from below using LED lights (LED Motion LED strips Eco 140+) with a luminous flux of 1742 lm m^−1^, operating at 1900 K colour temperature and consuming 19.2 W per meter of the LED strip. The LED light was filtered through a 650 nm cutoff low-pass acrylic filter (similar to Eckel et al, 2023). This setup created a bright background for the cameras, but not for the bees given their low sensitivity in this range of wavelengths, thereby maximizing the contrast of the bees' images on camera. Images were taken at a rate of 100 frames s^−1^, allowing the bees to be detected, tracked and their trajectories reconstructed in 3D. The exposure time of the cameras varied between 2 and 3 ms and was manually adjusted according to the natural lighting conditions in the room. This ensured that the dark bee and its long axis orientation could always be reliably detected against the bright background.

### Animal handling

We used four colonies of *Bombus terrestris* (Linnaeus 1758) from Koppert B.V. (Berkel en Rodenrijs, The Netherlands). Upon receipt, the bees were transferred into a custom-designed acrylic box measuring 24×24×40 cm^3^, which was draped with black cloth so that the nest was in the dark to imitate the bees’ natural environment. Within this enclosure, the bees were provided with pollen *ad libitum*, and had access to a 30% sucrose solution in a foraging area.

Foraging bees, i.e. bees undertaking flights between the hive and feeding chamber, were selected and individually marked. This process involved capturing the bee and immobilizing it within a marking tube. Subsequently, a numbered coloured plastic tag was fixed on the bee's thorax using resin, allowing for individual identification. Upon tagging, the bee was released back into the experimental setup near the nest entrance.

### Experimental procedure

After a 1 week habituation period, during which the bees had access to the foraging chamber by traversing the empty transit tunnel, we trained bees in two different cluttered environments and monitored their flight behaviour. On the outbound flight, bees flew through the empty transit tunnel into the foraging chamber. Bees that returned from the foraging chamber were sequentially directed into the experimental tunnel, where we recorded their inbound flight through clutter until they reached the entrance to the hive (see arrows in Fig. 1A). We trained two groups of bees in two different environments.

In the first environment, bees navigated through clutter composed solely of red acrylic objects, whereas in the second environment, 32 red objects along the central long axis of the tunnel were replaced by transparent ones (see Fig. 1B,C). It is important to note that the locations of the objects and thus their density and arrangement were the same for both environments, with variations only in the visual clearance of certain sections of the clutter between environments.

In instances where a bee failed to locate the tunnel exit within 5 min, the recording was stopped, and the bee was returned to the hive. Each bee underwent 15 trials. Overall, we recorded the flight behaviours of 35 bees (16 in the first environment and 19 in the second environment), sourced from four distinct hives.

### Trajectories

We recorded bee flight paths using a custom-written C++ recording software (available from our GitLab repository: https://gitlab.ub.uni-bielefeld.de/neurobio-public/mcam-suite). Our tracking procedure involved several steps. First, we established an image without bees by averaging the initial 100 frames of the recording. During recording, we implemented real-time background subtraction to identify bee movement. This process yielded cropped images showing only the differences between the current frame and background, along with corresponding spatial coordinates. Then we conducted image classification to retain only frames containing bees. To determine bee orientation, we utilized DeepLabCut (version 2.3.2) (Mathis et al., 2018) to identify and label the bee's head and abdomen positions based on our annotated dataset (accessible at https://pub.uni-bielefeld.de/record/2999609). The 3D flight trajectories were reconstructed through triangulation. When multiple synchronized cameras detected the bee simultaneously, we calculated its precise three-dimensional position within the tunnel environment. Complete documentation, including the code for the recording software, camera calibration protocols and trajectory reconstruction algorithms, is available from our GitLab repository (https://gitlab.ub.uni-bielefeld.de/neurobio-public/mcam-suite). For post-processing, we filtered the trajectories with a second-order lowpass filter and a cutoff frequency of 2 Hz.

For analysis of flight characteristics, we excluded single trials where bees were walking through the clutter. For flight path similarity analysis, trajectory preparation involved removing the initial and final 25 cm to mitigate artefacts arising from the flight's start and end. To analyse whether experienced bees choose similar flight paths through clutter, we considered directed trajectories from the fifth trial onwards. Because of the choice of similarity measures (see below), we needed to exclude trajectories where bees flew back and forth in the tunnel.

### Data analysis and statistics

To assess the similarity of individual flight paths across consecutive trials, we computed a similarity score utilizing distance functions. By comparing trajectories within each experimental group with each other using the Hausdorff distance, a distance matrix was generated. The Hausdorff distance is known as a geometry-based measure, representing the spatial similarity between two trajectories. A small distance value means that every point of either trajectory is close to some other trajectory points (Salarpour and Khotanlou, 2019). We calculated the similarity index by dividing the standard deviation of distance values among directed trajectories of the same bee by the standard deviation of distance values among randomly selected trajectories of the population of bees. For a reliable measure, we repeated this procedure 1000 times per bee (bootstrapping) and plotted the mean for each bee. A one-sample Wilcoxon signed-rank test was used to determine whether the median similarity index differed from 1, indicating greater similarity among individual bee trajectories compared with randomly selected ones.

To explore to what extent the bees optimized their flight behaviour through clutter with increasing experience, we analysed flight time, variability of flight speed, variability of lateral position, path sinuosity, and average and variability of minimal distance to objects. The flight time describes the time passed between the bee entering and leaving the experimental tunnel. The path sinuosity was obtained by dividing the length of the actual flight path by the length of the direct path between the tunnel entrance and exit. The closer the resulting value is to one, the more direct is the flight path of the bee. The variation of flight speed is described by the standard deviation. The variability of the lateral position is given by the standard deviation of positions along the *y*-axis of the tunnel during a flight. For the average and variability of the minimal distance to objects, we calculated the closest distance for each object passed by the bee during the flight and calculated the mean minimal distance and standard deviation per flight.

These flight characteristics were highly relevant for bees, especially when flying through cluttered terrains. Using (generalized) linear mixed models (gmler package and R version 1.4.1) with trial number and experimental condition as fixed effects and bee ID as a random effect, we assessed the impact of experience and environment on these flight characteristics. Furthermore, we assessed differences in flight characteristics between groups before and after training using a Wilcoxon rank-sum test with Bonferroni corrected *P*-values for multiple tests. Detailed statistical results, data and code are available from our GitLab repository (https://gitlab.ub.uni-bielefeld.de/m.jeschke/navigating_in_clutter_how_bumblebees_optimize_flight_behaviour_through_experience/-/tree/main).

## RESULTS

### Trajectories of bees differ between environments and level of experience

**Fig. 2.**
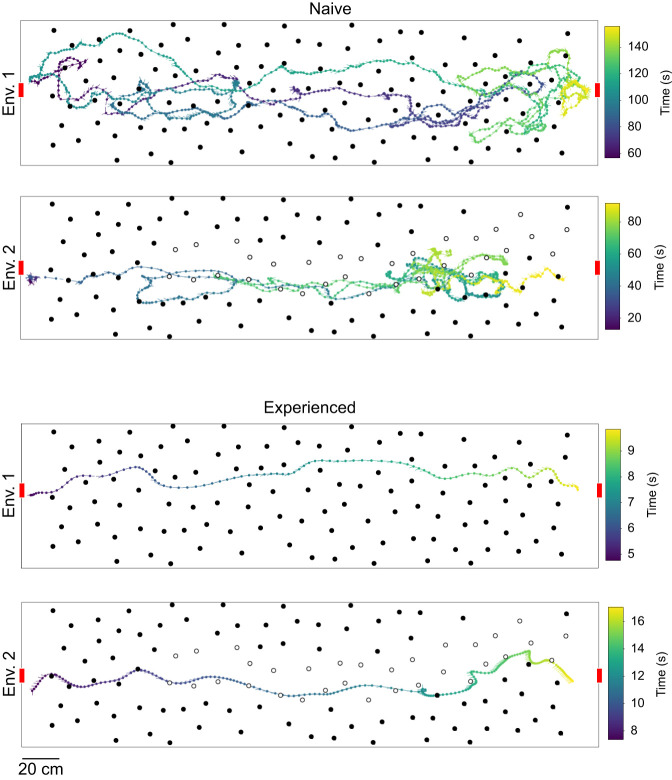
**Example trajectories of naive and experienced bees in both environments.** Each panel shows one example trajectory. The bees are flying through the tunnel starting at the exit of the feeding chamber (left) to the entrance of the hive (right). The tunnel entrance and exit are indicated by red rectangles. The trajectories show the position and orientation of the bees. The time is colour coded. Objects are shown as circles. Open circles refer to transparent objects.

We uncovered distinct behavioural patterns between naive and experienced bees as they navigated the cluttered environments (see Fig. 2). During their initial trial, naive bees in both environments showed exploratory behaviour, with indirect flight paths characterized by frequent back-and-forth movements within the tunnel. Surprisingly, we observed that collisions with objects were rare in both environments, regardless of whether some of the objects were transparent or not. Although the transparent objects were less salient and therefore should have been harder for the bees to detect, the bees were proficient at steering clear of collisions. Naive bees tended to spend a considerable amount of time within the tunnel before returning to the hive. In contrast, experienced bees showed a much more directed behaviour, displaying direct flight paths and efficiently crossing the cluttered area within a notably shorter time in their final training trial.

**Fig. 3.**
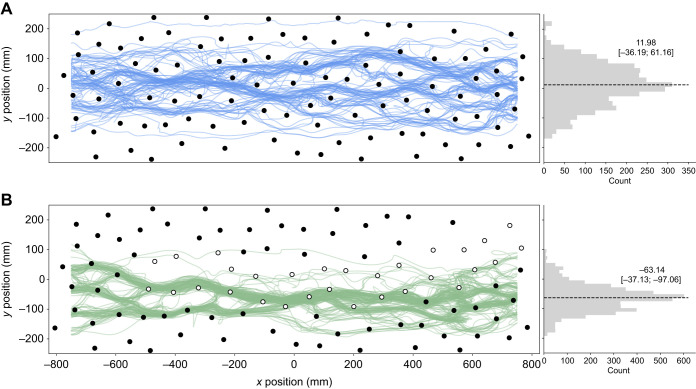
**Directed trajectories from the 5th trial onwards of all bees in each environment.** (A) Environment 1 (*n*=105); (B) environment 2 (*n*=155). The left subplot shows the trajectories of bees flying through the clutter. The object positions are indicated by filled and open circles for dark and transparent objects, respectively. The right subplot shows a histogram with the distribution of *y* positions. The dashed line indicates the average lateral position (median [interquartile range]).

**Fig. 4.**
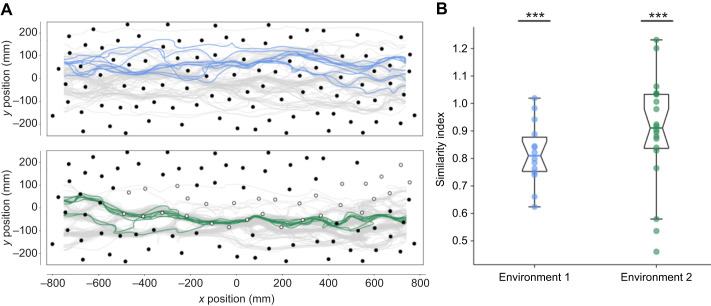
**Similarity of flight paths of individual bees.** (A) All directed trajectories from one example bee of environment 1 (top) and environment 2 (bottom) are shown in colour. All directed trajectories of the respected group are shown in grey. Dark and transparent objects are represented as filled and open circles. (B) Similarity index for both environments (environment 1 *n*=16, environment 2 *n*=19). The similarity index describes how similar trajectories of individual bees are compared with randomly selected trajectories of the population. Asterisks represent results of a one-sample Wilcoxon signed-rank test (***P<0.001).

After only a few training trials, bees showed direct flights through both environments. However, we observed that the paths taken differed if salient objects were replaced along the tunnel by transparent ones. Trajectories of bees in environment 1 (see Fig. 3A) occasionally clustered together to similar paths but were spread across a large part of the tunnel's width while maintaining distance to the walls. The median lateral position of these trajectories was close to the midline of the tunnel but with a large variability (median 11.98 mm, interquartile range [−36.19 mm; 61.16 mm]). Surprisingly, we found that trajectories in the environment with transparent objects (see Fig. 3B) often converged and bees flew more frequently through the right half of the tunnel with a median lateral position of −63.14 mm [−37.13 mm; −97.06 mm]. This side bias may indicate a potential asymmetry in the setup that was not immediately apparent. Notably, when bees were trained in mixed environments containing both transparent and coloured obstacles (see Figs 3 and 4), their trajectories tended to stay close to both types of obstacle. This suggests that bees may adopt a different flight strategy when navigating environments with obstacles of varying detectability, potentially treating transparent objects, which are more difficult to detect, similar to salient ones to ensure safe passage through the cluttered space.

Comparing the trajectories of individual bees across consecutive trials revealed that they often flew along a similar path (see Fig. 4A). The intra-individual difference of directed trajectories was smaller than the inter-individual difference of trajectories across the entire population. The similarity index, which compares trajectories of the same bee with randomly selected trajectories from the same environment, was significantly smaller than 1 in both environments (one-sample Wilcoxon signed-rank test; environment 1: median= 0.80, *P*=6.1×10^−5^; environment 2: median=0.91, *P*=1.9×10^−6^). This indicates that the trajectories of individual bees were more similar to themselves across trials than those of other bees within the population (see Fig. 4B).

Overall, we conclude that bees quickly learned to efficiently cross different cluttered environments and travelled along similar paths as they got more experienced in clutter.

### Bees optimize flight characteristics with increasing experience

**Fig. 5.**
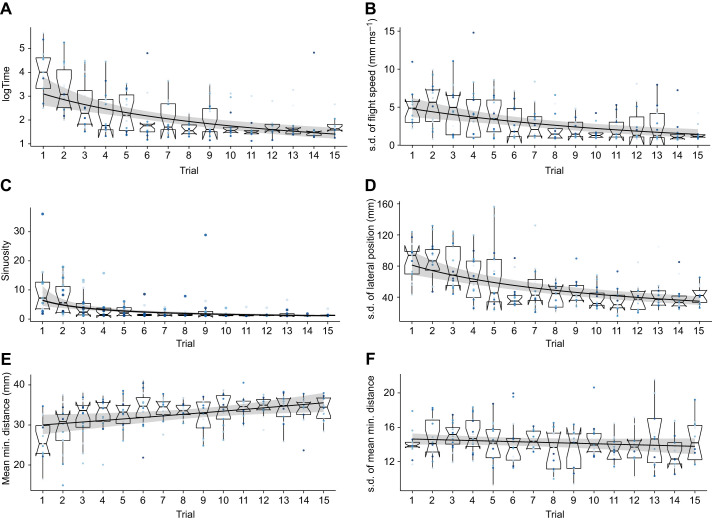
**Effect of increasing experience on flight characteristics of bees in environment 1.** (A) Time (in s) to cross the clutter. (B) Variability of flight speed in clutter. (C) Sinuosity of flight paths in clutter. (D) Variability of lateral position in clutter. (E) Average distance to objects in clutter. (F) Variability of distance to objects in clutter. Each subplot shows the development of one characteristic with increasing experience. Boxplots (median, upper and lower quartiles and 1.5× interquartile range) and individual coloured points show measured data (*n*=203), whereas the fit (black line) reflects the results of the generalized linear mixed model (GLMM) performed for the statistical analysis and the 95% confidence interval (grey shaded area). For more details of the statistics, see https://gitlab.ub.uni-bielefeld.de/m.jeschke/navigating_in_clutter_how_bumblebees_optimize_flight_behaviour_through_experience/-/tree/main.

**Fig. 6.**
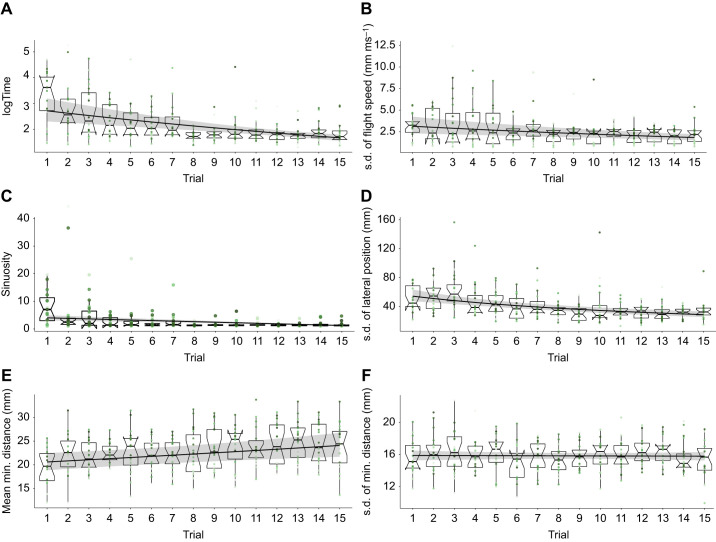
**Effect of increasing experience on flight characteristics of bees in environment 2.** (A) Time (in s) to cross the clutter. (B) Variability of flight speed in clutter. (C) Sinuosity of flight paths in clutter. (D) Variability of lateral position in clutter. (E) Average distance to objects in clutter. (F) Variability of distance to objects in clutter. Each subplot shows the development of one characteristic with increasing experience. Boxplots (median, upper and lower quartiles and interquartile range) and individual coloured points show measured data (*n*=286), whereas the fit (black line) reflects the results of the GLMM performed for the statistical analysis and the 95% confidence interval (grey shaded area). For more details of the statistics, see https://gitlab.ub.uni-bielefeld.de/m.jeschke/navigating_in_clutter_how_bumblebees_optimize_flight_behaviour_through_experience/-/tree/main.

We analysed the development of selected flight characteristics with increasing experience in both environments. Bees that flew through the dense tunnel with only salient objects optimized most of the analysed flight characteristics with increasing experience (see Fig. 5). They decreased the median time they needed to cross the tunnel over consecutive flights from 54 s to 5 s as well as the sinuosity of the flight paths. Moreover, their variability in flight speed decreased with increasing experience, indicating that more experienced bees varied their flight speed less during their flight and therefore performed less braking and acceleration manoeuvres while crossing the tunnel. The decrease in variability in the lateral position in the tunnel showed that bees used less than the whole width of the tunnel while they flew when they got more experienced with the clutter. The increase in the average minimal distance the bees kept to the objects during their flight suggested that they became more successful in avoiding obstacles. However, we observed no effect of experience on the variability in distance they kept to the objects in clutter. Bees that flew through a cluttered environment consisting of transparent and salient objects showed similar trends in optimizing their flight with increasing experience in clutter (see Fig. 6).

Although experience in clutter seemed to be an important factor influencing the flight characteristics, it might not be the only one. There may also be individual differences in how bees respond to such situations, such as variations in flight strategies or learning capabilities. To test whether, in a given environment, individual bees optimize flight characteristics differently, we included bee ID as a random effect in our generalized linear mixed models (GLMMs) and compared model performance with and without the individual bees as a random effect. Notably, GLMMs that included the bee ID as a random effect performed better than models without a random effect, suggesting that individual bees differed in optimizing the investigated flight characteristics in both environments (see tables 2 and 4 in https://gitlab.ub.uni-bielefeld.de/m.jeschke/navigating_in_clutter_how_bumblebees_optimize_flight_behaviour_through_experience/-/tree/main). This also corresponds to the finding that inter-individual variability between trajectories was larger than the intra-individual variability (see Fig. 4).

To determine whether the level of experience or the dense environment or both is a significant predictor for the flight characteristics, we fitted GLMMs with trial number and environment as fixed effects to the combined data of both environments. We fitted models with either the trial number or the environment, or both as a fixed effect and compared model performance using a likelihood-ratio test. We found that models for all characteristics performed worse when we removed the trial as a fixed effect, indicating that the level of experience is a significant predictor for these flight characteristics (see Table S1 for statistics). Moreover, we found that the environment is a significant predictor for the variance in lateral position, average minimal distance and variance in distance to objects, but not for the flight time, variance in flight speed and path sinuosity.

### Effect of the environment on novice bees

We found the performance of the bees in terms of flying through a cluttered flight tunnel to depend on the experimental conditions and in particular on whether some of the salient objects had been exchanged for transparent ones. This applies at least to some, though not all, of the flight characteristics analysed. Comparing the performance of bees in their first and last trial for all characteristics between environments reveals at which level of experience the environment has an effect on the flight behaviour.

**Fig. 7.**
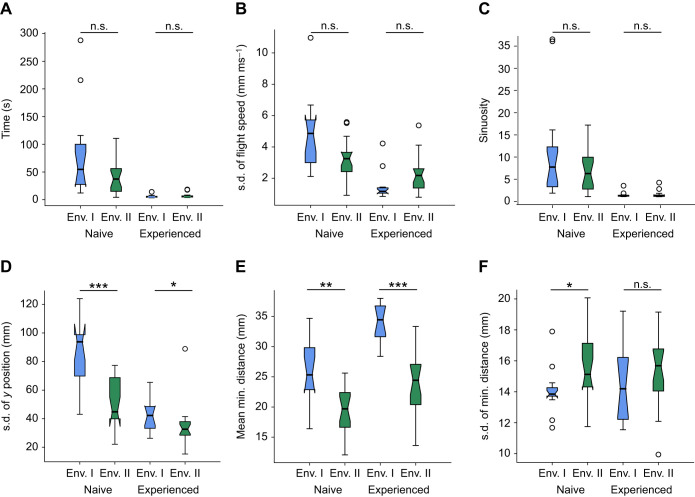
**Comparison of flight characteristics for naive and experienced bees between environments.** (A) Time to cross the clutter. (B) Variability of flight speed in clutter. (C) Sinuosity of flight paths in clutter. (D) Variability of lateral position in clutter. (E) Average distance to objects in clutter. (F) Variability of distance to objects in clutter. Each panel shows a flight characteristic of naive bees on their first training trial (environment 1 *n*=13, environment 2 *n*=19) and experienced bees on their 15th trial (environment 1 *n*=12, environment 2 *n*=19) as boxplots (median, upper and lower quartiles and interquartile range). Within the same level of experience, we compared characteristics between environments using a Wilcoxon rank-sum test. *P*-values (asterisks) are corrected for multiple tests (*P<0.05, **P<0.01, ***P<0.001).

The flight time and variability in flight speed showed no difference between environments in naive and experienced bees (see Fig. 7A,B). Bees reduced both their flight time and the variability of their flight speed with increasing experience. However, the presence of visually less dense areas in the environment did not affect either naive or experienced bees in terms of the time they required to traverse the clutter or the variability in their flight speed. In addition, we found that path sinuosity decreased with increasing experience but was not significantly different in naive bees or experienced bees between different environments (Wilcoxon rank-sum test for naive bees: *t*=1.0596, *P*=0.2893; Wilcoxon rank-sum test for experienced bees: *t*=−0.1726, *P*=0.8629) (see Fig. 7C).

However, the environment influenced where the bees flew within the clutter and how much their lateral position in the tunnel varied (see Fig. 7D). The variability in lateral position was 54% smaller for naive bees in the partly transparent clutter (Wilcoxon rank-sum test: *t*=3.5108, *P*=0.0004), and also experienced bees varied their lateral position 23% less when they were trained in the partly transparent clutter (Wilcoxon rank-sum test: *t*=2.3522, *P*=0.0186).

While these results might indicate that bees can cross a partly transparent clutter without problems of flying in between visually less salient obstacles, we observed that they nevertheless coped differently with salient and transparent objects (see Fig. 7E,F). Naive bees kept 31% more distance to objects in the fully visible clutter than bees that flew through the partly transparent cluttered environment (Wilcoxon rank-sum test: *t*=3.4340, *P*=0.0005). With increasing experience, bees in both environments increased the average minimal distance they kept to objects in their environment. At the end of the training, experienced bees in fully visible clutter kept on average 40% more distance to objects than experienced bees in the partly transparent clutter (Wilcoxon rank-sum test: *t*=4.3394, *P*=1.4285×10^−5^). Interestingly, the variability in distance they kept to objects was 15% smaller for naive bees in the fully visible clutter compared with the second environment (Wilcoxon rank-sum test: *t*=−2.4364, *P*=0.0148), but was not different between environments for experienced bees.

In summary, our results revealed that environments with differently salient objects significantly influence bee flight behaviour. Notably, naive bees navigating our cluttered environment maintained closer proximity to objects and showed reduced lateral position variation when encountering transparent objects.

## DISCUSSION

By having bumblebees repeatedly fly through two different cluttered environments and challenging their collision avoidance abilities by confronting them with either only conspicuous objects or partially transparent objects, we were able to demonstrate their navigational abilities in dense terrains. Our results show that bees rapidly learn to navigate successfully in an obstacle field and adapt their flight paths while progressively optimizing flight characteristics. As they gained experience in cluttered spaces, bees in both environments flew along increasingly similar trajectories. Surprisingly, bees that travelled through an environment containing largely transparent objects were also able to avoid collisions and optimized their flight with increasing experience, although the transparent objects in the environment provided less salient information for successful collision avoidance.

### Avoiding collisions with objects of different salience

Bees from both test groups successfully navigated the cluttered environment. Contrary to our prediction that transparent objects would hinder navigation, bees avoided all obstacles and maintained safe distances throughout their flights.

Even the edges of transparent objects probably produced subtle brightness contrasts, which through the bees' motion could generate optic flow patterns on their retinas – providing spatial information for navigation (Egelhaaf, 2023; Li et al., 2017). Nearby objects generate larger optic flow than distant ones, and expanding patterns signal approaching obstacles, enabling the bees to estimate distances and perform collision avoidance manoeuvres in a similar way to navigation through more conspicuous objects (Bertrand et al., 2015; Ravi et al., 2022). Although bees maintained safe distances from all objects, the shorter distances observed near transparent objects compared with the solid ones suggest that they perceive transparent objects as less visually prominent (see Fig. 7).

### Possible underlying mechanisms of route formation in clutter

An analysis of individual trajectories reveals that bees not only learn to cross cluttered spaces on relatively direct routes but also tend to follow remarkably similar paths in subsequent trials. This intra-individual consistency highlights the bees' ability to refine their navigation strategies through practice. The similarity of individual flight paths across trials was also observed on much larger spatial scales or different ecological contexts, such as traplining in foraging bumblebees (Lihoreau et al., 2010) or route formation observed in foraging ants in natural environments (Mangan and Webb, 2012). Besides insects, other flying animals also follow idiosyncratic routes while navigating through an obstacle course. Birds prefer to fly through wide passages in flight tunnels and select quick and safe routes (Bhagavatula et al., 2014). The established flight paths stay robust, even when a novel object is introduced (Karmaker et al., 2020).

The route formation we observed in our experiments might be a result of following a global direction combined with spontaneous collision avoidance manoeuvres, which is a rather reflexive behaviour and largely independent of experience or of route learning based on experience in the cluttered environment. These two aspects are not mutually exclusive and are difficult to distinguish experimentally. Therefore, we conducted experiments with non-transparent and transparent objects and challenged collision avoidance mechanisms and learning mechanisms to identify their relative role for bumblebees when optimizing their flight behaviour in cluttered terrains.

Previous modelling studies have shown that collision avoidance algorithms coupled with a goal direction can produce goal-directed trajectories similar to those observed in navigating insects in clutter and the formation of a small number of routes through the simulated dense environment (Bertrand et al., 2015). As collision avoidance is a reflexive behaviour and largely independent of experience, the bees in our experiment would only need to learn the goal direction, i.e. the exit of the tunnel. After an exploratory flight, we would expect the bees to show direct flights through the cluttered environment in subsequent trials. However, we instead observed that bees progressively optimized their flight and needed multiple trials before they showed direct flights through the clutter. Moreover, challenging collision avoidance mechanisms by using transparent objects in our environment led to a similar optimization of flight characteristics with increasing experience. Therefore, learning mechanisms appear to play an essential role in route formation in dense environments. This conclusion is also supported by modelling studies investigating visually guided obstacle flight in birds (Lin et al, 2014; Ros et al., 2017). Pigeons flying through vertical poles not only steer towards the nearest opening but also show a flight direction biased towards larger visual gaps, suggesting that it is not just simple collision avoidance that plays a role during navigation through cluttered environments.

As we observed that the bees progressively optimized their flight with increasing experience in clutter, we assume that learning is involved in the process of flight optimization and route formation. Several papers have shown that insects can establish a spatial memory and strategically use learned visual landmarks for navigation (Collett et al., 2013). It is not entirely clear what visual features the bees learn and use for navigation in our paradigm, such as panoramic views, single objects or object constellations as landmarks. For future experiments, we could test bees in a rearranged environment after they have been trained to cross the clutter in a direct manner to further disentangle the role of learning versus collision avoidance. If route learning plays a crucial role, we would expect the bees to slow down and show less straight flight paths in a newly arranged environment. If the bees purely rely on a collision avoidance strategy, we would expect a rearranged cluttered environment not to have a significant effect on their flight characteristics.

### Optimization of flight behaviour in clutter

By carefully controlling for the bees' level of experience in our cluttered environments, we could show that bumblebees can optimize their flight behaviour. This becomes evident by the reduction in time to cross the clutter, the decreased variability in flight speed, lower sinuosity of flight paths and distance to objects observed in both experimental groups.

While there has been little research about how flight characteristics evolve with increasing experience in cluttered terrains, tunnel experiments with experienced bees have been used a lot to study insect flight. Therefore, we can compare only our results of experienced bees with those of previous studies on flight characteristics of bumblebees.

In comparison with experiments using empty tunnels with a similar tunnel width to ours, our bees in the cluttered tunnels fly with a mean flight speed of 0.368±0.046 m s^−1^ on average 20–46% slower and show a larger variability in flight speed (Baird et al., 2010; Linander et al., 2015). Our bees fly with a 20–24% faster flight speed compared with those that fly through a tunnel with obstacles in their frontal field and have to perform lateral manoeuvres to avoid them (Burnett et al., 2020; Crall et al., 2015). However, the bees in our experiment fly 56% slower than bees that are only confronted with obstacles in their lateral visual field that form a corridor the animals can fly through (Lecoeur et al., 2019). Therefore, we can strengthen the conclusion that flight speed in cluttered environments is highly influenced by the position of objects in the visual field.

Bees flying through an empty tunnel were shown in several studies to closely control their lateral position and fly along the midline with little variation in their lateral position (Chakravarthi et al., 2017; Linander et al., 2015; 2016). However, with objects positioned along the walls in the tunnel, the variability of lateral position decreases with increasing object density (Lecoeur et al., 2019) and bees increase the precision with which they control their lateral position.

When our bees are trained to fly through a homogeneous cluttered environment of similar looking visually conspicuous objects, they fly on average in the centre of the tunnel but also show a large variation in their lateral position, probably due to the presence of objects in their lateral but also frontal visual field. Interestingly, a cluttered environment with transparent objects and, thus, visually less dense areas led the bees to shift their average lateral position towards areas containing conspicuous objects and they showed less variation in their lateral position. The drop in variation in their lateral position may be explained by the aggregation of flight paths to an area with salient objects they can easily detect and by the reduced distance they kept to objects in this environment.

The comparison of results of different experiments clearly shows that flight characteristics are influenced by environmental factors such as the presence and placement of objects in the environment. Future experiments could investigate the potential influence of further environmental factors, such as the object density or spatial arrangement, on flight behaviour, which would help us to understand how flight characteristics evolve in different cluttered terrains.

### Conclusion

Our study reveals that bumblebees effectively optimize their flight behaviour in cluttered environments through experience. In addition to collision avoidance, which constrains the way they traverse the clutter, they use their experience to adapt to the visual challenges posed by the clutter. Our findings highlight the ability of bees to refine the characteristics of their flight paths in response to environmental complexity. Notably, individual bees develop consistent trajectories over time, suggesting the formation of spatial memories and learned navigation strategies.

## Supplementary Material

10.1242/jexbio.250514_sup1 Supplementary information
