# Supplementary material for: Navigating in clutter: how bumblebees optimize flight behaviour through experience
Source: J Exp Biol. 2025 Jul 25;228(15):jeb250514. doi: 10.1242/jeb.250514 (PMC12319407; doi:10.1242/jeb.250514)
Supplement: Supplementary information [file jexbio-228-250514-s1.pdf]

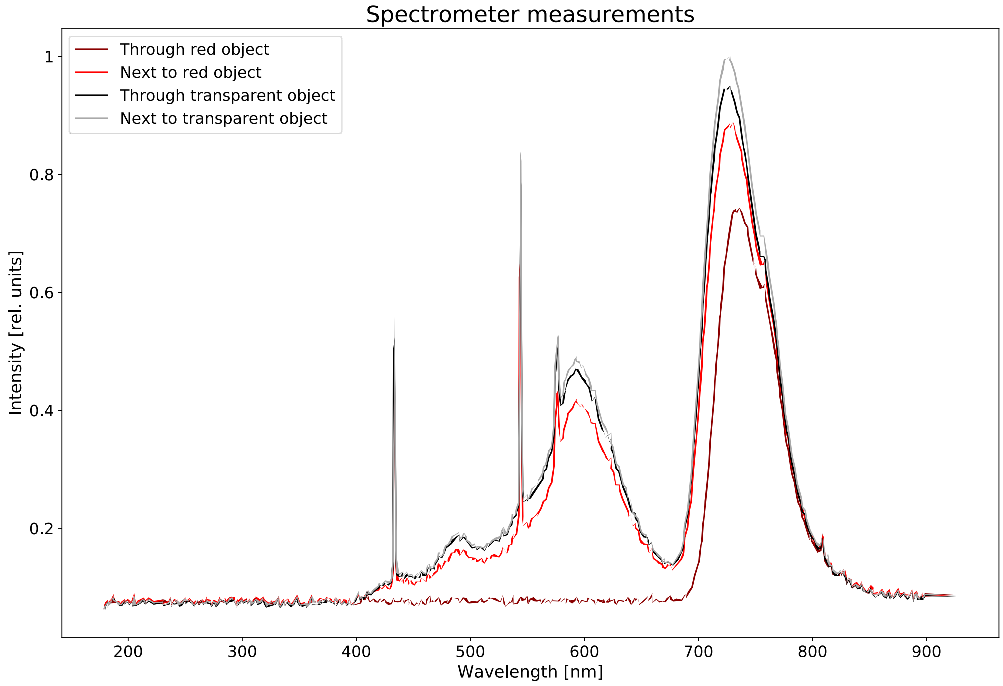

Fig. S1. Spectrometer measurements of different parts in the experimental setup. Using the same light conditions as during the experiments, we measured the light reflected by a dark red object (dark red curve), by a transparent object (black curve), the light inside the tunnel next to a dark red object (red curve) and the light inside the tunnel next to a transparent object (grey curve). Note that the red acrylic that was used to build the objects blocks light below 650nm. Therefore, the objects appear dark for the bees. The transparent objects allow all the light to pass through.

Table S1. Likelihood-ratio-test results to compare the goodness of fit for models including the trial number, the environment or both as fixed effects for combined data of both environments

	#Df	LogLik	Df	Chisq	Pr(>Chisq)
(1) log(time)~trial+environment+(trial|beeid)	7	-395.95			
(2) log(time)~trial+(trial|beeid)	6	-396.32	-1	0.746	0.387
(3) log(time)~environment+(trial|beeid)	6	-407.55	-1	23.19	$1.46e^{-06}$
(1) speedstd~trial+environment+(trial|beeid)	7	-799.70			
(2) speedstd~trial+(trial|beeid)	6	-799.89	-1	0.375	0.539
(3) speedstd~environment+(trial|beeid)	6	-810.64	-1	21.89	$2.89e^{-06}$
(1) sinousity~trial+environment+(trial|beeid)	7	-898.37			
(2) sinousity~trial+(trial|beeid)	6	-879.20	-1	74.33	0.000
(3) sinousity~environment+(trial|beeid)	6	-916.37	-1	36.00	$1.97e^{-09}$
(1) ystd~trial+environment+(trial|beeid)	7	-2047.66			
(2) ystd~trial+(trial|beeid)	6	-2053.18	-1	11.03	0.0008
(3) ystd~environment+(trial|beeid)	6	-2064.52	-1	33.72	$6.34e^{-09}$
(1)distobj_mean~trial+environment+(trial|beeid)	7	-1309.30			
(2) distobj_mean~trial+(trial|beeid)	6	-1323.60	-1	28.06	$8.87e^{-08}$
(3) distobj_mean~environment+(trial|beeid)	6	-1314.99	-1	11.39	0.0007
(1) distobj_std~trial+environment+(trial|beeid)	7	-1051.64			
(2) distobj_std~trial+(trial|beeid)	6	-1061.22	-1	19.17	$1.19e^{-05}$
(3) distobj_std~environment+(trial|beeid)	6	-1049.57	-1	4.14	0.041

Journal of Experimental Biology • Supplementary information
